# On the Artificial Production of Stupidity in Schools

**Published:** 1859-04-01

**Authors:** 


### Art. II.—ON THE ARTIFICIAL PRODUCTION OF STUPIDITY IN SCHOOLS.

It is related of a learned judge, that he once praised a retiring witness in the following words: "You are entitled to great credit, sir. You must have taken infinite pains with yourself. No man could naturally be so stupid."

We cite this well-worn anecdote because it contains, probably, the earliest public recognition of the principle which the title of our article is intended to convey. Existing in all ages of the world, in all conditions of life, and described by a copious vocabulary in every language, stupidity is something which it has never been possible to ignore or to forget. The fact of its all-pervading presence, its vitality in the most different climates and scenes, has tended to convince mankind of the necessity of an evil which they have never failed to perceive; and which has served, from time immemorial, as a subject for the lamentation of the wise, and a basis for the calculations of the designing. The lessons of proverbial wisdom, the results of hasty generalization, and the daily experiences of life, all point out, or seem to point out, that stupidity is inseparable from the existence of the human race; and that it must appear, not in every individual, but in many individuals of every community. It follows, that the persons in whom the phenomenon is most conspicuously manifested are regarded with something of the compassion which attaches to physical infirmity; and enjoy, in a certain degree, the power of blundering, with the privilege of being exempt from punishment. "Davus sum, non Œdipus;" and the happy Davus eludes responsibilities which a wiser man would be compelled to bear. "Le jour va passer, mais les badauds ne passeront pas;" and the *blagueur* need never doubt that he will find dupes when he requires them.

We have long entertained a conviction that this passive acquiescence in stupidity, as an ultimate fact of human nature, and this confident expectation of its unmitigated recurrence in each succeeding generation, are founded upon errors of considerable practical importance. By directing attention to causes that are remote, they induce forgetfulness of those which lie at every man's door; and, by bringing into prominence the stupidity which is irremediable, they lead us to neglect examination of that which may be prevented.

In truth, the varieties of hebetude are numerous. It must be admitted that some of them are displayed by persons whose intellects are obscured by organic defect, "native and to the manner born," in the nervous apparatus,—by continuing deficiency, or excess, in the composition or quantity of the circulating fluid; and it is probable that, in many cases of this nature, the scalpel, or the microscope and test-tube, would fail to disclose the cause of the infirmity. Inherited diathesis, or hereditary disease, may doubtless weaken the faculties of the mind, as they evidently weaken the physical powers of the body, and may produce effects varying in degree from idiocy to mere dulness of apprehension. We are far from saying that in these instances stupidity can neither be alleviated by judicious, nor confirmed by improper treatment; but we indicate them as affording a substratum of truth to popular prejudices touching the general invincibility of the state in question, and as giving evidence of its centric rather than eccentric origin.

But leaving this subdivision of the stupid entirely out of consideration, and remarking, by the way, that the word stupidity is misapplied when used to denote the mere absence of brilliant talent, we would call attention to the large class of persons who are dull and obtuse, not by reason of any probable congenital deficiency, nor by an unfair comparison with great wits or geniuses, but by comparison with what the individuals themselves clearly ought to be—with what they would have been had their faculties been developed in the right way. And this comparison is not so difficult as it may appear; for the simple reason that the human capabilities do not greatly depart, save in exceptional cases, from the standard of mediocrity. Among a score of men taken at random, but approaching to equality in point of conformation, we may observe that physical strength or endurance will vary only within very narrow limits: there being perhaps a single athlete, or a single weakling, and a remainder composed of individuals whose powers are not precisely on a level, but nearly so.

Let us suppose, however, that among the twenty men there were a certain number who had been employed from their early years in pursuits calculated to produce muscular vigour and hardihood, and who had observed all rules and precautions likely to ensure to such pursuits their most favourable effects. It is certain that, whatever differences might exist amongst themselves, these men would surpass all their competitors. Bendigo, the champion of the prize ring, was one of a triple birth, and was the weakliest child of the family in which, by reason of diligent training, he is now the strongest man.

So universally has this principle been recognised and acted upon, that in every barbarous or half-civilized community, or under all circumstances which give an unquestioned superiority to bodily strength, we may find evidences of special care to foster and increase it. The "games" obligatory upon the little Spartans, the exercises of "gentle youth" during the age of chivalry, the description given by Mr. Catlin of the early training of the American aborigines, are all instances in point; and all show the recognition, under circumstances widely dissimilar, of the principle that the powers of the human organism are bestowed only *in posse*—to be developed by culture, or to dwindle under neglect.

The state of physiological knowledge permits us to lay it down as an axiom that what is true of one system or apparatus, among those given to man, must also be true, *mutatis mutandis*, of the rest. Without in the least degree failing to perceive the dependence of the higher faculties upon a spiritual nature, we must also perceive their dependence, during this life, upon the qualities of their material organs, the nervous centres; and the dependence of these qualities upon the laws which regulate nutrition and cell growth. We are therefore entitled to assume, *à priori*, that, precisely as the methods of the trainer raise the physical powers of his disciples to the highest point attainable by each organism, so analogous methods would raise the intellectual powers in the same manner and degree. The conclusion which may be formed by reasoning is not unsupported by experience; but the masters of the art are few, and the examples of their skill are rare.

In an age of bodily repose, with nearly all locomotion artificial, with money as the principal purveyor, and with A 22 to remove the necessity for self-protection, it is not surprising that men are careless about their physical powers, and think them hardly worth the trouble which their full cultivation would entail. Under circumstances in which strength of arm and fleetness of foot have afforded the chief sources of security, or have opened the most direct paths to renown, there has never been an approach to indifference about the means by which these qualities might be attained. If physical education be now almost wholly neglected, it is because the utility of its results has been diminished by the progress of civilization.

But this age of bodily sloth and weakness is also, it must be remembered, an age of intellectual activity and strength. The wide diffusion of knowledge, the facilities for travel, and the application of philosophy to the comforts and conveniences of life, have increased a thousandfold the value, to each possessor, and to the whole human race, of the perceptive and conceptive faculties of the mind. Every one who observes the facts within his sphere, and reflects upon them, may find the key to some as yet unopened door in the temple of nature, or may excogitate results calculated to increase the happiness of man. The career that offers itself to the intellect surpasses immeasurably all that has ever been offered to the corporeal powers; and it might therefore reasonably be expected that intellectual development would be the subject of the same foresight now, which the development of the corporeal powers was wont to call forth in former days. It might be expected (although strength and activity of limb are left to come of themselves, under the unaided influence of that playful restlessness of the young which provides against muscular atrophy) that the training of the higher faculties of the mind into due vigour and perfect symmetry would be carefully studied as a science, and diligently practised as an art. It might be expected that the mechanism of observation and of thought, the nature and order of the processes by which, chiefly, wealth, and power, and fame are to be acquired, would be the subjects of an attention corresponding to the degree in which wealth, and power, and fame, are prized. It might be expected that every one—the poor man to the extent of his means, and the rich man to the extent of his knowledge—would seek to confirm and strengthen in his offspring the qualities by which the world is ruled.

That the endeavour would not be fruitless, we have abundant evidence. Reasoning from an analogy which cannot fail, we find that the human organism scarcely ever approaches, under the influence of casual impressions or spontaneous acts, to anything like the full measure of its powers. The average athlete is but the corporeal perfection of the average man—a perfection the result of labour, and which the common games of youth or pursuits of manhood are insufficient to produce or to maintain. The most striking example upon record of the physical predominance of one class of men over all others with whom they came in contact, was furnished by the Roman legionaries, in the days of the Roman conquests. It may be explained by the system which trained each legionary like a gladiator; and it disappeared as that system was relaxed and abandoned. "Nulla enim alia re videmus populum Romanum orbem subegisse terrarum," says Vegetius, "nisi armorum exercitio, disciplina castrorum, usuque militiæ." The citizens of Rome, as such, could possess no natural superiority over, and in some cases not even an equality with, the inhabitants of the countries they subdued; but the citizens of Rome were trained to the exercises and formed to the discipline of war. Their physical powers were improved to the utmost, and they were inured to every variety of labour, fatigue, and hardship. The world has not witnessed a school of mental education upon a method so excellent, or upon a scale so grand; but the proverbial sagacity of the Jesuits, and the proverbial erudition of the Benedictines, may be cited to show that the mind will respond, always in some degree, and often vigorously, to a stimulus greater than that which is supplied by the usual events of life. It has been well said that nature throws forth her able men as a salmon does its spawn, but produces her great ones as a lioness does her cub—singly, and at rare intervals. Whenever the want of an able man is felt and acknowledged, it is almost invariably supplied from among a limited circle of lookers-on, one of whom will find in the occasion a means of at once discovering and developing capabilities formerly dormant. The various persons whose duties have required them to undertake original investigations into the phenomena of physical science, have nearly always exhibited a remarkable intellectual growth as one reward of their exertions. They have become more cautious, more sagacious, more diffident than before; and there is not the slightest reason to suppose that they were, in the majority of instances, men of exceptional natural powers. On the contrary, the parallel facts connected with the muscular system, and the remarkable uniformity with which the faculties of reflection and judgment expand and strengthen under proper use, may conjointly be taken to prove that the ordinary life of civilized Europe does not develop either body or mind in a degree at all commensurate with their capacities for action. The cricket-field and the boating-club produce a certain amount of vigour and hardihood; but their most ardent votaries would be exhausted by the pastimes of a savage, or by the daily drill and duty of a soldier of old Rome. From the universities, and from schools of the first order, issue many men unquestionably of high attainments, and some of great and cultivated parts; but the aggregate of both classes may be said to have a point of resemblance to Brummell's finished cravat, and to suggest that a large number of "failures" have been quietly conveyed downstairs. In schools of an inferior kind, the attainments of the pupils are less conspicuous; and the existing state of mental education may be summed up in the earnest and weighty words of Professor Faraday, who declares that, "in physical matters, multitudes are ready to draw conclusions who have little or no power of judgment in the cases; that the same is true of other departments of knowledge; and that, generally, mankind is willing to leave the faculties which relate to judgment almost entirely uneducated, and their decisions at the mercy of ignorance, prepossessions, the passions, or even accident." The same authority says again, that "society, speaking generally, is not only ignorant as respects education of the judgment, but is also ignorant of its ignorance."

It must be conceded, we apprehend, that in the present day no man is called upon to undergo a course of severe physical training, or to exercise the muscular system to the acme of its powers. But it must also be conceded that there have been conditions of society which rendered such training the duty of every one, and in which it was enforced by a public opinion of the most rigid kind. We think that, in the times in which we live, the duty of mental cultivation is at least equally binding, and that its performance requires to be prompted by the same incentive.

For we are convinced that a very large proportion of the stupidity now existing in the world is the direct result of a variety of influences, educational and social, which operate to the prejudice of the growing brain, either by checking its development altogether, or by unduly stimulating the sensorium at the expense of the intelligence. In the former case, general obtuseness is the result; and in the latter, subjugation of the reasoning powers to the sensations or emotions. We are entitled to think these conditions strictly artificial; and to look upon them as distortions, analogous, in some respects, to the physical distortions of Hindoo fakirism.

The educational influence which, more than any other, is concerned in producing them, appears to us to be due to confusion of thought on the subject of those very distinct realities called knowledge and wisdom. While the prevailing weaknesses of the human mind—those apparent to the philosopher, and those also which are manifest to the vulgar—are alike due to want of wisdom, the efforts of ordinary instructors, and the general current of the events of life, are chiefly valued as they appear calculated to impart knowledge. It is not surprising that such should be the case, a great impulse having been given to education in this country at a time when the operations of the mind were not sufficiently understood to allow of a just discrimination between them. Learning, and (prior to the modern development of various branches of science) learning of one especial kind, was essential to the attainment of a position in which wisdom could be conspicuously displayed. It followed that every man whose wisdom was known to the public, was known also to be

"A scholar, and a ripe and good one;"

while the illiterate, whatever their natural powers, were almost compelled to remain among the "mute inglorious Miltons" of the community. Moreover, learning was a thing apparent and undeniable, easily perceptible to many who were unable to fathom its depths; while wisdom could only be recognised by the kindred wise, or in a fruition not always directly traceable to its causes. Hence, and in a manner not difficult to comprehend, arose a general impression that the acquisition of knowledge was the principal or even the only means of gaining wisdom; and this impression was confirmed by experience of the fact that mental development is frequently coincident with efforts to learn. The exact relation between the two is not easy to define, even with all the aid afforded by recent advances in psychology; but, in former times, it was the opinion of the most advanced educationists, that a certain routine of teaching afforded the best discipline for the growing brain, and that this routine, when aided by good abilities, was certain to produce the highest attainable results—so that men of moderate or inferior performance, who had received "a good education," were considered to be the failures of nature, and not of the preceptor. The hypothesis was most comfortable, serving to shift responsibility from tutors and professors, and to place it where it was borne without a murmur; while the necessary interval between the schools and life was sufficient to render obscure any possible connexion between bad teaching and eventual stupidity. During the universal prevalence of such principles as these, commenced a movement which was formerly described as "the march of intellect," but which was, more correctly, a march of schooling. Men of various calibre, and various degrees of learning, were cordially united in an attempt to elevate the masses by education. For this purpose they organized a scheme by which to pour forth knowledge like water, and, in carrying it into practice, they spared neither age nor sex. Cheap publications explained everything—in a manner to be comprehended by everybody. The fathers of England were taught (with diagrams) the philosophy of their daily duties; the mothers, of their household avocations. Even unhappy little children, struggling through the sands of school, were caught and engulphed by the advancing wave. The great and good promoters of the original measure were overwhelmed by the co-operation of innumerable amateurs, who expected to make learning universal, by addressing, to the untaught, condensed statements of scientific results, and who looked forward to a time when the intellectual vigour of the community would be gauged by the reports of the Society for the Confusion of Useless Knowledge, or by the sale of illustrated penny serials, as the material prosperity is at present by the quarterly returns of the Registrar-General. The idea seemed to be, that the diffusion of knowledge would act as a stimulant upon all minds of sufficient natural power, and would call forth their energies—would set them thinking, comparing, judging; and that the rest of mankind, those not vitalized by the potent influence, were to be regarded only as *caput mortuum*, unworthy of consideration in a philosophical sense, however formidable in point of numbers.

Notwithstanding the great and sudden illumination to which we have referred, there is no evidence of any remarkable advancement, any increase at all commensurate with the pains bestowed, in that cultivation of mind by which alone knowledge can be applied or rendered useful. In every rank, children are taught many things which were unknown to their forefathers; and the operations of the Committee of Council on Education have wrought a marvellous change in the position, with regard to learning, of the sons and daughters of the labouring poor. But school work cannot be correctly estimated by the results of the half-yearly examination; and requires to be tested more severely, and more truly also, by the events of life. The reports of Her Majesty's Inspectors, especially for 1855-6, are well calculated to direct attention to this view of the case; and they show that the improvement which was hoped for, nay, almost expected, as a result of teaching, has not yet been realized. The young adults who have passed through aided parochial schools do not present any marked superiority, either moral or intellectual, over others who have not had that advantage; and the learning acquired at these institutions would appear to be of the most transitory kind. The words (already quoted) of Professor Faraday, rendered doubly emphatic by the known and habitual caution of their author, may be taken as conclusive with regard to persons of a higher station; and the whole evidence appears to show that the reasoning faculties, in all classes of the community, are very imperfectly and insufficiently developed—imperfectly as compared with their natural capabilities—insufficiently when considered with reference to the extent and variety of information with which they are called upon to deal. We are compelled to seek for the causes of this deficiency in an educational system that makes no adequate provision for mental training; and we think that a brief review of the relations between the nervous centres and the impressions that form the basis of knowledge will enable us to point out the precise nature of the chief errors in existing practice, and to define the principles by adherence to which those errors might be obviated.

The first point to which we would call attention is the existence, in the young of the human species, of a distinctly *duplex* educability: depending upon distinct functions of the brain. It may be taken as conceded, we apprehend, by all physiologists, that the encephalon of man differs from that of other Mammalia chiefly by the super-addition of parts whose office it is to control the succession of ideas, and to determine the course of conduct. The powers of *re-collection*, comparison, reflection, and volition, are attributes essentially human; or, at least, are possessed by men in common with higher intelligences alone. The powers of sensation, ideation, and spontaneous remembrance, are possessed also by the lower animals; and are sufficient to explain all the particulars of their conduct.

It is manifest, therefore, that the education of a child may be conducted, in the direction, and to the extent, in which it is possible to educate a horse, a dog, or an elephant, without necessarily trenching upon, or at all arousing, any faculty that is distinctly human in its nature. The child, moreover, possesses an endowment, of a purely sensational or animal kind, in which brutes are deficient: namely, the power (subsidiary to the gift of language) to remember a great number of sounds, and to imitate them with facility; so that, just to the extent of this power, the sensational educability of the human race exceeds that of the lower animals.

It should be remembered, moreover, that the functional activity of the sensorial tract of the encephalon is an absolute necessity of animal existence; and that, in men and brutes alike, it is provided for by an energetic tendency to spontaneous development under the influence of its appropriate excitants. In what may be termed the natural life, a blind submission to the promptings of sensations, present or remembered, would, in all ordinary cases, supply the wants, or gratify the passions of man. It is only in life modified by human aggregation that these promptings require to be controlled by an exercise of will, guided by a prior exercise of judgment; and therefore, while Divine Providence has endowed the human race with sensational faculties that are called into vigorous action by daily wants, or by physical impressions from without, we may observe that the higher powers of the mind, in a great majority of instances, cannot be matured excepting by assiduous cultivation.

In this respect, however, there is probably a considerable original diversity between individuals; and we are much inclined to think that herein consists the chief cause of gradations of ability among persons who neither greatly surpass an average standard, nor fall greatly short of it. Observation teaches that it is far more easy in some children than in others to carry instruction beyond the sense-perceptions, and to call the intellect into activity; but it teaches also that the supposed difficulty often arises from an improper selection or application of the means employed, and is simply a failure to open a lock with a wrong key. The apparently dull child not unfrequently receives the necessary stimulus from a trivial circumstance, from a conversation, a book, or a pursuit, and may grow into a gifted man; while a parallel transformation may be accomplished, much later in life, under the influence of some new opportunity for action. It is possible that, in minds of the highest order, the intellectual faculties may possess the character of spontaneity which is commonly limited to the sensorial tract; but, in all ordinary cases, these faculties require to be excited in the pupil by their presence, and their activity, in the teacher.

The sensational and intellectual functions of the human brain are not only distinct, but also, in some degree, antagonistic, through the application of the ordinary law of nutrition to their respective organs. The portions of the encephalon that are most employed will receive the largest supply of blood, and will be the seats of the most vigorous cell-growth, precisely as the same rule will apply to the development of muscle; while on the other hand, a certain duration of disuse, or of restricted use, will occasion atrophic changes, and will be followed by that functional impairment which is a natural result of structural degeneration. It follows that men of the highest intellectual activity are often somewhat inattentive to impressions made upon their senses; and also that great sensational acuteness is often purchased at the cost of some torpor, as regards the operations of the judgment.

Upon testing the educational customs of the present day by even the most elementary principles of psychology, it becomes apparent that a very large number of children receive precisely the kind of training which has been bestowed upon a learned pig. There are scarcely any schoolmasters who have in the least degree studied the operations or the development of the mind (indeed it is only within a very few years that this study has borne any fruit of great practical utility); and those who have not done so cannot realize the existence of a kind of learning which is sensational alone. Indeed, it is more in accordance with ordinary preconceptions to refer brute actions to a process of reasoning, than to consider that any human actions are automatic. The truth is, however, that the first impressions made upon the consciousness of a child have a strong natural tendency to expend themselves through the sensorium; and usually do so, unless directed higher by the manner in which they are produced or maintained. For the purpose of such direction, time is an element of the first importance; and the idea which would be grasped by the intelligence after a certain period of undisturbed attention, will excite the sensational faculties alone, if that attention be diverted by the premature intrusion of something else that solicits notice. And while, in almost every child, the power of intelligent attention may be aroused by care, and perfected by perseverance, the natural inclination is towards a rapid succession of thoughts, variously associated, and remembered in their order without being understood. The faculty of comprehension, like all others, is a source of pleasure to the possessor, even in the first feeble attempts to bring it into exercise; and hence, as well as from the impulse given to nutrition, when once a habit of endeavouring to comprehend has been formed, although in very young children, it is not readily relinquished; but, on the contrary, is applied to the most unpromising materials.

In schools, however, under the stern pressure of the popular demand for knowledge, it is an extremely common practice to accumulate new impressions with greater rapidity than they can be received, even by children who have enjoyed the inestimable advantage of early domestic training towards the right employment of their higher faculties. The work laid down can often only be accomplished by means of the promptitude that is a chief characteristic of instinctive action. The child who uses his sensorium to master the sounds of his task, uses an instrument perfected for him by the Great Artificer. The child who uses his intelligence must perfect the instrument for himself, must grope in the dark, must puzzle, must catch at stray gleams of light, before his mind can embrace the whole of any but the simplest question. The former brings out his result, such as it is, immediately; the latter by slow degrees, often first giving utterance to the steps by which he is reaching it. The former is commonly thought quick and clever; the latter slow and stupid: and the educational treatment of each is based upon this assumption, widely as it is often at variance with the facts. The child whose tendency is to sensational activity should be held back; and be made to master the meaning of everything he is allowed to learn. He is usually encouraged to remember sounds, is pushed forward, is crammed with words to the exclusion of knowledge, is taught to consider himself a prodigy of youthful talent. The child who tries to understand his lessons should be encouraged, praised, supplied with food for thought of a kind suited to his capacity, and aided by a helping hand over the chief difficulties in his path. He is usually snubbed as a dunce, punished for his slowness, forced into sensational learning as his only escape from disgrace. The master, in many cases, has little option in the matter. Children are expected to know more than they have time to learn; parents and examiners must have show and surface, things only to be purchased at the expense of solidity and strength. A discreet teacher may often feel sympathy with the difficulties of a pupil; but the half hour allotted to the class is passing away, the next subject is treading upon the heels of the present one, the child must complete his task like the rest, and so a budding intellect may be sacrificed to the demands of custom.

Among the children of the educated classes the circumstances of domestic life usually afford to the intelligence an amount of stimulus which, if not of the best possible kind, is at least sufficient to compensate, in some degree, for the sensational work of school. The easy nursery lessons of the pre-scholastic age, the story-books of childhood, the talk of parents and friends, all furnish food for leisurely reflection, all serve to suggest those strange questions that are one chief evidence of thoughtfulness in the young. Minds thus prepared may often flourish, in spite of subsequent excessive teaching; and by forgetting nine-tenths of what has been learned, may find it possible to understand the rest.

In what are called "Elementary schools," however, those aided by the nation for the instruction of the children of the poor, we do not find this accidental provision against the paralysing effects of the prescribed routine. For the most part, the children have grown up like wild animals, excepting for the advantage of an occasional beating; and their nervous centres have received few impressions unconnected with the simplest wants of existence. Coincidently with an entire absence of intellectual cultivation, they usually display a degree of sensational acuteness not often found in the nurseries of the wealthy; and arising from that habitual shifting for themselves in small matters which is forced upon them by the absence of the tender and refined affection that loves to anticipate the wants of infancy. They go to school for a brief period; and the master strives to cram them with as much knowledge as possible. They learn easily,—but they learn only sounds; and seldom know that it is possible to learn anything more. In many cottages there are children who, as they phrase it, "repeat a piece" at the half-yearly examination. We say, from frequent experiments, that they will learn for this purpose a passage in any foreign language as easily as in English; or that they will learn an English paragraph backwards way, if told to do so; and that, in neither case, will any curiosity be excited about the meaning of the composition. In ordinary practice, the master explains what they repeat, saying, this means so and so; and the pupils have sufficient sensational acuteness to remember the sounds he utters, and to reproduce them when called upon. They do not usually understand what "meaning" is. An urchin may be able to say correctly that a word pointed out to him is an adverb or a pronoun, may proceed to give a definition of either, and examples of instances of its occurrence, and may produce an impression that he understands all this, when the truth is that he has only learned to make certain noises in a particular order, and when he is unable to say anything intelligible about the matter in language of his own. Or he may repeat the multiplication table, and even work by it, saying that seven times eight are fifty-six, without knowing what fifty-six is, or what seven times eight means. He knows all about seven or eight, not from schooling, but from the lessons of life, from having had seven nuts or eight marbles; but of the fifty-six, which is beyond his experience, he knows nothing. The nature of the mental operations of such children is perhaps as little known, to the teacher, to the vicar of the parish, or the kind ladies who take an interest in the school, as the nature of the mental operations of the inhabitants of Saturn. The adults distinctly understand a thing which they feel to be very easy, and do not know that any children *can* talk about it correctly without attaching an idea to their words. They often think the teaching satisfactory which enables the pupil to explain things in set phrases. They do not realize the possibility that the explanation may be as little understood as the statement which it explains—that it may be like the tortoise in the Hindoo myth, which supports the elephant, but which, requiring support itself, only removes the difficulty by a single step—that it may be a second unknown quantity balancing the first in the equation $x = y$. Such, however, instead of bare possibilities, are too frequently actual results.

The best recorded illustration of such sensational learning is given by the Rev. Mr. Brookfield, H.M.'s Inspector, in his official report for 1855-6. Mr. Brookfield called upon two children, aged about eleven years, "who did their arithmetic and reading tolerably well, who wrote something pretty legible, intelligible, and sensible about an omnibus and about a steam-boat," to write down the answers of the Church catechism to two questions. It must be observed that they had been accustomed to *repeat* the Catechism during half an hour of each day, in day-school and Sunday-school, for four or five years, and the following is what they wrote :—

"My duty toads God is to bleed in him to fering and to loaf withold your arts withold my mine withold my sold and with my sernth to whirchp and to give thinks to put my old trast in him to call upon him to onner his old name and his world and to save him truly all the days of my lifes end.

Again—

"My dooty tords my Nabers to love him as thyself and to do to all men as I wed thou shall do and to me to love onner and suke my farther and mother to onner and to bay the queen and all that are pet in a forty under her to smit myself to all my gooness teaches sportial pastures and marsters to oughten mysilf lordly and every to all my betters to hut nobody by would nor deed to be trew in jest in all my deelins to beer no malis nor ated in your arts to kep my ands from pecken and steel my turn from evil speak and lawing and slanders not to civet nor desar othermans good but to lern laber trewly to git my own leaving and to do my dooty in that state if life and to each it his please to god to call men."

Again—

"They did promis and voal three things in my name first that I should pernounce of the devel and all his walks pumps and valities of this wicked wold and all the sinful larsts of the flesh."

A story equally characteristic has recently appeared in the *Glasgow Commonwealth.* It relates that a traveller in one of the western islands of Scotland was assailed by a pert and communicative little boy, who offered to repeat to him the names of all the capitals in Europe, and who did so without error or apparent difficulty. The traveller, being a person of inquiring mind, rather sceptical as to the value of the lad's acquirements, asked him if he knew the name of the island he lived in (Skye) ; and, to prevent any misapprehension of the question, it was repeated in Gaelic, but no name was forthcoming. He knew the name of the parish, and of almost every capital in the world, but not of the island he lived in. The traveller then ventured another question, "Now, my lad," quoth he, "you have told us the names of nearly all the capitals in the world; is a capital a man or a beast?" "It's a beast," said the boy, quite decisively.

The paraphrase of the Catechism recorded by Mr Brookfield has been often quoted ; but we have thought it worthy of reproduction here, if only on account of the observations which that gentleman has made concerning it. He remarks, very justly, that the error is not a mere matter of spelling, not a phonetic expression of ideas that are understood, but that it involves absolute non-apprehension of the meaning of the passages. He is startled by the discovery of this non-apprehension, and thinks it traceable to the almost obsolete language of the Catechism, while he believes in the general intelligence of the children, as shown by their power of writing what was not nonsense about certain objects. We cannot, of course, express an opinion otherwise than from the facts before us ; but we are strongly tempted to believe that these objects were familiar to the children out of school, and that their knowledge of them was gained from experience rather than from teaching. We have observed similar non-apprehension, over and over again, of matters expressed in current phraseology; but school teachers and managers seldom observe it, because they seldom look deep enough. They are mostly unacquainted with the complexity and extent of sensational operations in the young; they have scarcely ever been accustomed to analyse the acts of the mind, and they think they have probed the depths of intellectual consciousness before they have even approached the surface. Working with the intelligence themselves, and feeling more or less a sense of discomfort in connexion with what is obscure, a *besoin de comprendre*, a necessity to puzzle, they have no experience of that tranquil resting upon remembered sensations which is, we believe, the most frequent result of their tutorial labours.

We have already referred incidentally to a learned pig, and to the parallelism between its training and some kinds of human education. Persons familiar with the tricks taught to animals are aware that these may all be described as muscular actions performed each consecutively to its proper signal. On hearing the finger nails of the master click together, the animal does something in obedience to the sensation; nods its head, or shakes its head, or stands erect, as the case may be. It has no idea that the nod is an affirmation, or the shake a negation, and probably has no thirst for knowledge about the matter, being content to play its part correctly, and to escape the whip. In the case of children, the medium of communication is different, and the kind of response is different; but the faculty in action is commonly the same. The words of the pig's master are mere by-play, intended to amuse the audience, and the signal is conveyed by other sounds. The words of the human teacher or examiner, his questions for instance, are the signals to the child, each requiring its appropriate answer; but like the signals to the pig, they are aural sensations, capable, as such, of producing muscular action through the medium of the sensorium alone. The responses of the child are in words—that is to say, in sounds that he has been taught, and that he remembers, but of which he need not understand one iota in order to repeat them, any more than the pig need understand the affirmative or negative character of its nod or shake. In the human species articulate speech is an act precisely analogous to locomotion, requiring the combined and harmonious working of several muscles, and the guidance of sense, but in no way essentially connected with the intelligence; and the child may make the right noises in the right order, just as the pig does not nod its head when the signal requires it to be shaken.

A general idea of the facts which we have endeavoured to state was conveyed to the public, many years ago, by a phrase now almost forgotten. Educationists found, by experience, that children managed to retain sounds without meaning, and they called the process " learning by rote." Books, pamphlets, and speeches bore witness to the practical inutility of such learning, and were full of suggestions for improving upon it. But these suggestions, to the best of our recollection of them, did not go to the root of the matter, and were mainly based on the assumption that learning by rote was characterized by some sort of deficiency only, and not by a radical error in the kind of impression made upon the pupil. It was not distinctly stated, or commonly conceded (although often implied in phraseology), that the action of the child's mind was of a nature essentially distinct from that which it would be the object of a wise instructor to excite; and the cause of the error was mainly sought in teaching not carried far enough to be beneficial, or not continued sufficiently long to produce permanent results. We conceive that the recent development of nervous physiology entitles us to maintain that learning by rote is at once the effect and the evidence of operations limited to the sensorial ganglia; and that such operations have no tendency, however they may be complicated or prolonged, to excite those functions of the cerebrum which are the peculiar attributes of humanity.

Our brief remaining space must be devoted to an examination of the effects of sensational learning, both as it exists, *pure et simple*, in most schools for the poor, and also in the form, more or less modified, which may be found in other institutions.

Physiologically speaking, the effect of purely sensational learning will be to stimulate the nutrition and increase the vigour of the sensorial tract at the expense of neighbouring and related organs. As we have seen, the sensorium has a natural tendency to predominance in the encephalon; and this tendency will be increased in every way, absolutely by direct excitation, and relatively by neglect of the intellect and volition. The sensations by which the stimulus has been given will not be long remembered, being superseded by fresh ones arising out of events, as the apparatus of the gymnasium would be superseded by the instruments of actual conflict. With the exception of being perhaps able to read with labour, and to write with difficulty, the pupils must not be expected, six months after leaving school, to possess any traces of their "education" beyond an invigorated sensorium and a stunted intelligence.

The transitory nature of the so-called learning is abundantly shown by the reports of her Majesty's Inspectors. One of these gentlemen, with admirable naïveté, italicises the following question:—"*To what purpose in after life is a boy taught, if the intervention of a school vacation is to be a sufficient excuse for entirely forgetting his instructions?*"

Now, when it is remembered that present sensations are the source of the least exalted kinds of animal gratification, and that sensations, either present, or remembered, or conceived, when combined with a feeling of pleasure or pain, constitute the emotions which so powerfully influence human conduct, it must be admitted that the sensorium is at least the seat of development of those passions and propensities which society, for its own good, is compelled to keep in check, and which every consideration of right teaches individuals to subdue. When, therefore, we reflect upon the operation of predominant emotions in producing, among other evils, chorea, hysteria, epilepsy, and insanity, or when we consider the aggregate of misery produced, especially among the lower orders, by the unbridled indulgence of various appetites, we cannot altogether concur in the propriety of a system of education which has a direct tendency to raise the source of these emotions and appetites to an undue and unnatural prominence in the organism.

As evidence of the stunted intelligence of children withdrawn from elementary schools, we have to offer the simple theory of the process, the testimony of H.M.'s Inspectors, and the results of personal observation.

Under the first of these three heads it is only necessary to point out the effect of habitual sensational activity in rendering the pupil content with sense perceptions. The *besoin de comprendre*, the love of knowledge inherent in all minds, will not survive the continual and energetic repression of a teacher, who says practically to the children—"You must learn this lesson, or work this sum by rule; but you must not take time enough to understand what you are doing." The class thus treated will not only cease to think about their tasks, but they will leave school prepared to act without thought in all the relations of life. Few of them, under any training, would be eminent in philosophy; but fewer still, perhaps, would have been left by nature the utterly unreasoning animals that they frequently become.

The testimony of her Majesty's Inspectors, as contained in their annual reports, will hardly admit of quotation within the limits of our space. It is apparent that these gentlemen endeavour to discover the best features of the system which they superintend; and their most damaging admissions are often obscured by an unconscious circumlocution arising from a constant balancing of the praiseworthy against the blameable. The educational blue book for 1855-6 we have already mentioned as containing more reference than many others to the real efficiency of schools; and having thus indicated a source from which abundant materials for the formation of a correct judgment may be drawn, we will content ourselves with the following very brief citations:—

The Rev. F. Watkins says:

"On all sides you hear of the little regard paid by young people to parental authority, of the great love of dress, and carelessness about running into debt, of pleasure-seeking at cost of time, money, and character, above all, of the increase of drunkenness, that fruitful mother of all vices. It is impossible to hear all these constantly reiterated statements, and to be convinced of their general accuracy, without feeling that, whatever may have been earnestly and rightly attempted towards the education of the working classes, there is but little yet really done."

The Rev. W. J. Kennedy says:

"I think there is truth in the statement that those who leave our national schools deteriorate intellectually rather than improve."

Dr. Woodford says:

"Boys who were employed in extracting square and cube roots, and who were pretty successful in bringing out the right result * * * not only could not express, but had no idea of what was meant by the term *root* in relation to that of square or cube."

The test of personal observation must always be difficult to apply, and liable to the fallacies which invalidate conclusions drawn from a limited number of instances. But, in our own experience, we have met with so many examples of what may be called habitual non-reflection in young people who had been, six months before, among the most glib and fluent pupils at a sensational school, that we fancy we can recognise a kind of stupidity thus induced, and that we can readily distinguish it from anything at all similar that is purely natural. The former variety bears a strong general resemblance to animal instincts, as they are sometimes displayed under circumstances which must obviously defeat their purposes (thus a captive beaver will construct an useless dam in his place of confinement), so as to prove to demonstration that the creatures exhibiting them have no conception of the objects which, in a state of nature, they blindly but unerringly attain. Our readers may easily note for themselves examples of conduct similarly aimless, or may hear of them from any lady who has ever attempted to train, as a household servant, a girl from the village school. The examples will mostly resolve themselves into this, that directions given are acted upon, like instinctive impulses, "prior to reflection." The particular cases in point are mostly trivial; but we cannot abstain from placing upon record that a budding domestic, being told by her mistress to put wire gauze covers over various eatables on the shelves of a larder, piled all the covers, Ossa upon Pelion, over one dish, and left the remaining ones at the mercy of the flies of August. Unquestionably, great pains must have been bestowed upon her.

Apart from such consequences to the children, there are others, not unworthy of note, which affect the parents or the community. Educationists raise their voices and wail, because the attendance of the pupils is irregular, and their removal commonly premature.

In other words, the labouring classes do not cordially respond to the invitations which are held out to them for the benefit of their offspring. They use the schools for their own purposes only. They regard them as places of refuge for infants and young children, serving to take them out of the way of the busy housewife, and to shelter them from the perils of the street; but, in an overwhelming majority of cases, the teaching received enters scarcely at all into the account. It does not impart anything which untaught parents can themselves appreciate, neither does it develope the general intelligence in such a way as to excite their admiration or command their sympathy. When boyhood or girlhood is attained, the children are permitted to leave school, in some cases that they may indulge in the luxury of idleness, in some that they may respectably follow creditable employments, in some that their earnings may assist (with or without urgent need for such assistance) in the maintenance of the family. There are probably very few instances in which the departure is sincerely regretted either by the child or its parents.

We say "sincerely" because the school often represents a powerful interest which the parents think it necessary to conciliate, even at the expense of a kind of duplicity which too frequently enters into their daily life. It is not uncommon for a mother touchingly to deplore the necessity for her son's removal, and to tell the schoolmaster, (with a corner of her apron in her eye) that the employer of the father has insisted upon the services of the boy;—when, in reality, the work has been eagerly sought, and the employer prevailed upon to countenance the deception.

It is hardly necessary to advance any argument to prove the general indifference of the industrious poor with regard to schooling, except this, that they will make almost any sacrifice, undergo almost any privation, to obtain that which they really value. If they valued schooling, if they thought that one year more, or two years more, would be truly useful to their children, there are thousands who would cheerfully endure cold and hunger rather than allow the children to be deprived of the advantage. There have been many instances of such self-denial, exercised in furtherance of other laudable objects; but not one, within our observation, for the sake of school. We feel convinced, if elementary schools are ever raised out of their present dreary routine of sensational teaching, if they ever succeed in awakening the intelligence of a fair proportion of their pupils, that the eagerness of the poor for education will speedily keep pace with the liberality of the rich in providing it; and that the nation will have the satisfaction of being able to point out results, as well as to grumble over payments.

We turn from this tempting theme, this brief vision of a scholastic Utopia, in order to consider the processes and results of Dr. Grindall, the presiding genius of Blunderbore House for Young Gentlemen.

These processes and results are, upon the whole, what might be expected from a teacher who ignores the great truth that cultivation of mind is necessary to the assimilation of learning; and who imagines that the introduction of compressed facts will mechanically expand the intellect. Upon this last false principle Master Thompson, in this nineteenth century, and in the ninth year of his age, is forcibly and tyrannically inducted into various kinds of knowledge: in the hope that all the teaching and lecturing and cramming, all the scraps of science, bundles of facts, odds and ends of common things, Greek verbs, Latin verbs, German verbs, French verbs, Scripture history, ancient history, modern history, natural history, rules of syntax, rules of arithmetic, rules of algebra, and rules of conduct, the propositions of Euclid and the theory of ventilation, the rationale of catarrh and the law of storms, that all these several matters will eventually, like the talk of S. T. C., "converge in light;" and coherently illuminate a full-grown Thompson, possessed of sufficient ballast for his sails, sufficient parts for his attainments, and sufficient brains for the application of his learning.

The Young Gentlemen, it must be remembered, have not spent their pre-scholastic years in making dirt pies in a gutter. Had they done so, had the instructions of Dr. Grindall been the first that were ever afforded them, the normal elementary school result would have followed as a matter of course;—the sensational learning, the dense unreflecting stupidity. But young gentlemen, for the most part, have tender and loving mothers, whose pleasant task it has been to make every sense a door leading to the intelligence. The intellect, thus called into activity, can seldom be wholly crushed beneath instruction. Sometimes (as shoots of ivy will lift or rend a rock), it even springs into luxuriant growth, pushes away the cumbrous obstacles of so-called learning, finds for itself the aliment required for its support, and animates the pupils who are the pride of the school, who gain its honours, receive its rewards, support its reputation at the universities and in the world. Much more frequently, it is condemned to an etiolated and weak existence, as may be seen in the numerous boys in whom the desiderated convergence has not occurred; but whose minds are productive of chromatic aberration, fringing transmitted facts and arguments with blue, red, or yellow, according to variations of temperament or character. In these boys, after years of costly and pretentious teaching, one may observe such mental and general habits, and such a store of really available information, as they might have gained at the humble commercial academy of a country town. Is it that they represent the proportion, whether large or small, of pupils who are so organized as to receive no commensurate benefit from the best kind of education, who are incorrigibly idle, or incurably dull, or, in fact, the failures of nature rather than of the preceptor? We think not. Nature, we believe, is seldom such a bungler. She is the *alma mater*;—Art the *injusta noverca.*

We should be disposed, on the whole, to seek the rationale of the Blunderbore House failures rather in a partial and misdirected training of the intelligence, than in its complete suppression. The pupils mix intellectual and sensational acts, not in their proper relations with each other, but in a jumble. Comprehension is brought to bear upon everything that is easy; while a difficulty of any kind is committed to the safe keeping of the sense perceptions, and the explanation of it is only remembered. Hence arise a habit of resting upon imperfect knowledge, and a habit of loading the memory by the aid of faulty associations; and these habits, in their turn, are the sources of the lively superficial stupidity which is so common among the better classes. The sufferers from it form that great public to whom are addressed the Morisonian system of pathology and therapeutics, and the elaborately argued advertisements of Norton's Camomile Pills. Everything that follows "because" is to their minds an explanation; everything that has an antecedent is to their minds an effect. Their creed is that all questions lie in a nutshell; and, according to Professor Faraday, their shibboleth is "it stands to reason." On this ground they would placidly maintain against Owen the existence of the sea-serpent. For their especial behoof bubble companies are formed; and upon their weaknesses innumerable Barnums thrive. Their deficiency is chiefly this,—that having been permitted from childhood to do many things superficially and with inexactness, they have forfeited the power of arranging their ideas with precision, or of comparing them with caution. They can therefore scarcely be said to possess any assured convictions, or rooted principles of conduct; but, nevertheless, they are ready to decide in all controversies; and are "wiser in their own conceit than seven men who can render a reason."

The cause of such educational errors we should express in the single word—empiricism. For successive ages teachers had no guide but experience; and the results of this experience appeared to defy generalization. The almost self-evident proposition, that the training of the mind should be guided by an analysis of its powers, was brought into early disrepute by the conditions under which such analysis was attempted. The men engaged in it, learned, patient, laborious, profound, reached the limit of discovery by the method of reflection long before the method of observation was disclosed to them. Too exclusively metaphysical, they wanted a link to connect them with the material world. Like the children of Israel, they were wandering in a wilderness before they entered the promised land. Their advanced messengers had not yet returned, bringing of the fruits that were hereafter to reward their labour. Foiled in their advance by a barrier that seemed impassable, they were tempted to waste their energies in the invention of technicalities and the multiplying of verbal distinctions. Under such circumstances the science and its professors were too broad a mark to escape the shafts of satire; and thus, even at the present day, there are scars to show the wounds which those shafts have made.

During the last few years, however, the dark portions of this much contemned pursuit have received unexpected illumination from the study of the nervous centres. The painstaking researches of Bell, Marshall Hall, and less conspicuous fellow-labourers, endowed with value and stamped with currency by the lofty generalizations of the living philosopher who has so long been *facile princeps* among all inquirers into the functions of the nervous system, have already produced a psychology that is available for practical purposes, and that promises to increase daily in importance. In the meanwhile education has spread enormously; but educators persist in traversing the broad old road. The larger the field for their efforts, the more conspicuous becomes the poverty of their results. At one time, learning by rote was the great obstacle; and they attacked, as the last difficulty in their path, what was but the first aspect of a Proteus. At present (with the scheme of National Education all but a confessed and palpable failure; with numerous individuals in all ranks displaying powers developed, late in life, by circumstances, but never suspected before; and with a waste of the national intellect that may possibly be equivalent to the daily loss of a century's progress), the office of preceptor is still confided to persons who have never bestowed a single thought upon the faculties or the mechanism of the mind, and who cannot distinguish between sensational and intellectual action, if the former be veiled by the smallest complexity. And, as a crowning absurdity, a reverend Inspector of Schools towers, like Milton's chaos, above the fray; and proposes a panacea, based upon an error that was exploded, sixty-seven years ago, by the pen of Dugald Stewart!

We must not conclude the present article without mentioning the kinds of reform that appear to be most urgently required; although we propose, upon an early occasion, to consider this portion of the subject in detail.

In elementary schools for the poor, there should perhaps be nothing attempted, except to give a capacity for self-education. For this purpose the mechanical difficulties of reading and writing should be thoroughly overcome, and the teacher should bear in mind that his pupils require from him the first stimulus to the intelligence. Instead of the little ones being left to pupils or monitors, they should be the especial charge of the master himself; and their first efforts to learn and understand should be promoted with the most assiduous care and the most untiring patience. The tracks of sensation and intellect diverge; and the child will follow that into which he is guided at the outset of his journey.

In the ordinary time allotted to schooling, the several divisions of the scheme of elementary instruction are mere *ignes fatui*, which it is hopeless to pursue. The children cannot learn Geography, or History, or half a dozen other matters. But by sacrificing these they might learn to read with facility and pleasure, to write, to work and comprehend a simple sum. They might also be made to feel the gratification inseparable from an exercise of the understanding; and, if they did so, the library would complete what the school was compelled to leave unfinished.

The schools for classes higher in the social scale could only be improved upon similar principles; but the home training of the pupils, and the longer time devoted to them, must always afford facilities for combining a good deal of instruction with the education. The recent middle class examinations show clearly that teachers have failed in the former as decidedly as they have in the latter: and this result need not excite surprise. For instruction without mental education must necessarily resemble the plum-pudding that was made in Paris; and for which everything was remembered, *except the cloth.*

Towards the carrying out of any improvement, however, the first step must be to demand from teachers either a knowledge of mental philosophy, or, at least, of a scholastic art founded upon the principles which mental philosophy would inculcate. We believe this demand must inevitably be made in process of time; but we feel also that it would be greatly promoted if the medical profession would recognise, and strive to impress, the distinct bearing of physiology upon the development of the mind, as well as upon that of the body.

The practical difficulties which it is easy to foresee, all resolve themselves, pretty clearly, into one. An inquiry after intelligent and intelligible teaching has not yet issued from the public. They are content with something else. Whenever this contentment ceases, the means of supply will spring out of the want. And until then we would urge, upon individual parents, that they may accomplish much by encouraging, in their little ones, a spirit of curiosity and a habit of comprehension. Whether the fire of intellect shall blaze, or smoulder, will depend, in many cases, upon the manner in which it is kindled; and this kindling is among the things that can be done, most effectually, under the mild influences of Home.

---

### Art. III.—THE METHOD AND STATISTICS OF SUICIDE.

